# Supplementary material for: Mucin and Agitation Shape Predation of Escherichia coli by Lytic Coliphage
Source: Microorganisms. 2023 Feb 17;11(2):508. doi: 10.3390/microorganisms11020508 (PMC9966288; doi:10.3390/microorganisms11020508)
Supplement: Supplementary file 1 [file microorganisms-11-00508-s001.zip › microorganisms-2122487-supplementary.pdf]

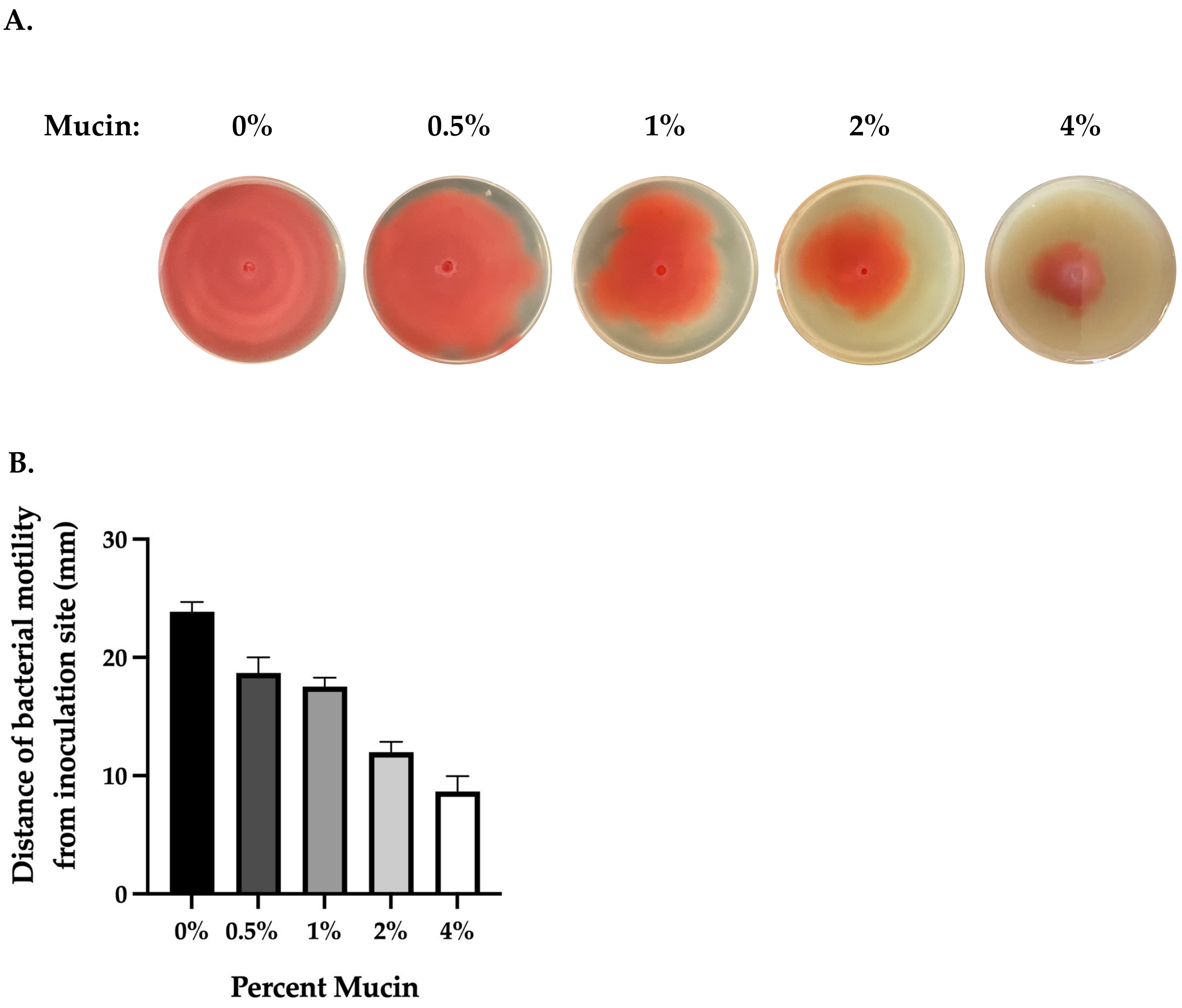

Supplementary Figure S1. *Increasing mucin concentration hinders bacterial motility.* (A) Motility of *E. coli* K803 as indicated by bacterial metabolism of Tetrazolium (red color) after overnight incubation of inoculated motility plates containing increasing concentrations of mucin. Bacteria (10^4) was inoculated in the center of the plate. (B) Graphical representation of mean distance swum by K803 in motility plates containing increasing concentrations of mucin. Bars are mean ± SEM.

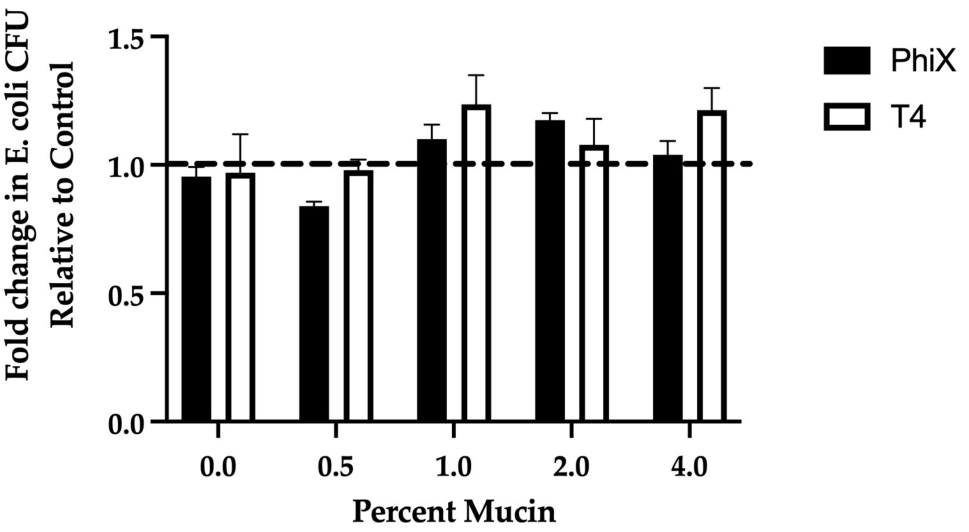

Supplemental Figure S2. *After 3 hour incubation, bacteria-phage interactions do not result in measurable bacterial lysis at MOI 0.1.* In-tube growth of K803 in all mucin concentrations tested (0.5%-4%) following 3 hrs incubation with either PhiX (black bars) or T4 (white bars) at an MOI of 0.01. Values are normalized to growth of bacteria alone in LB (1; represented by dashed line).
